# Pandemic influenza 2009 on Réunion Island: A mild wave linked to a low reproduction number

**DOI:** 10.1371/currents.RRN1145

**Published:** 2010-01-19

**Authors:** Philippe Renault, Eric D'Ortenzio, Florence Kermarec, Laurent Filleul

**Affiliations:** ^*^Regional office of the French Institute for Public Health Surveillance; ^†^Regional office of French Institute for Public Health Surveillance (Institut de veille sanitaire, InVS) Saint-Denis, Réunion Island, France; ^‡^Environmental Health Department of the French Institute for Public Health Surveillance, Saint-Maurice, France and ^§^Regional Office of the French Institute for Health Surveillance

## Abstract

We studied the epidemic trend following the introduction of the pandemic A(H1N1) 2009 in the subtropical Réunion Island. There, the pandemic wave started from week 30 and lasted until week 38, with an estimated attack rate of 12.85 % for symptomatic infections. The best estimate for the initial reproduction number was Ri = 1.26 [1.08; 1.49]. It results that the herd immunity necessary to stop the epidemic growth is of the same magnitude than the attack rate. Thus, a second wave before the 2010 austral winter seems unlikely, unless a viral mutation.

## Introduction

The reproduction number and the generation time are among the most important epidemiologic parameters designed to estimate the transmissibility of an infectious disease. Since the emergence of the influenza A pandemic H1N1, the first studies published suggested values around three days for the generation time and between 1.4 and 3.2 for the reproduction number[1]. However, these studies, which gave rather high estimates for the reproduction number, have been conducted on the available data from Mexico or from outbreaks in closed settings. Therefore, its value might have been overestimated. More recently, a Canadian study found a lower estimate of 1.31 for the reproduction number on the general population of Ontario between April 13 and June 20, 2009[2]. On Réunion Island, pandemic influenza A (H1N1) 2009 took place during the regular seasonal period for influenza and was followed by a monitoring system tested and alerted well in advance. These conditions seemed optimal to allow a fairly accurate estimate of the reproduction number in the specific context of this island.

## Methods

Réunion Island is a southern hemisphere subtropical Island of 810,000 inhabitants located in the south-western Indian Ocean (Fig.1). 

**Figure:**
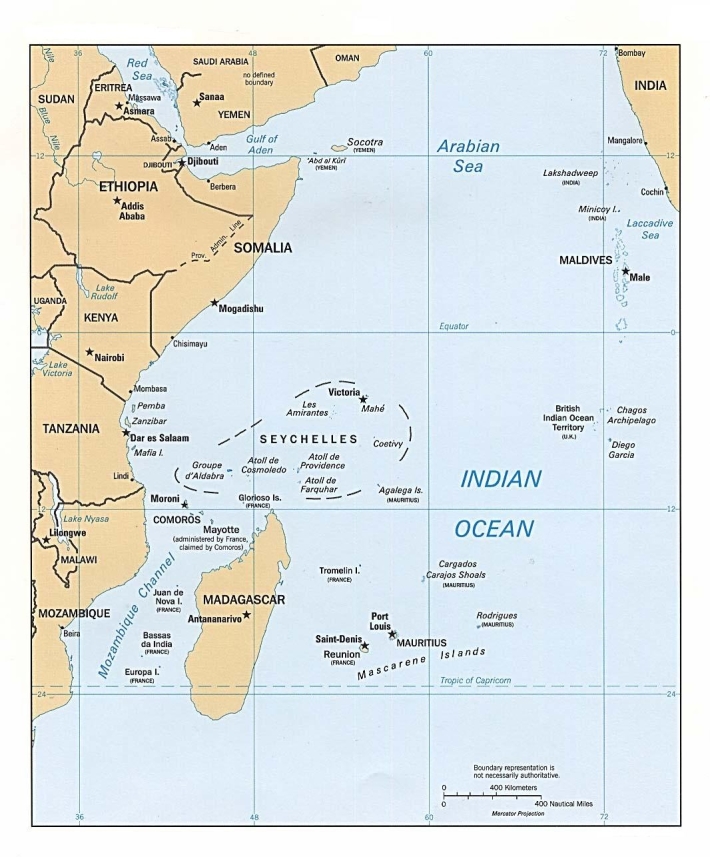


Figure 1: Map of the Western Indian Ocean featuring Réunion Island. Reprint by permission of Intute Publishers (Oxford, United-Kingdom)

On Réunion Island, the enhanced influenza surveillance implemented by anticipation since May 2009[1], detected the first case of influenza A pandemic H1N1 on 5 July in a traveller returning from Australia and the first autochthonous case was reported on 22 July. From this date onward, the number of consultants for influenza A pandemic H1N1 was estimated from the data of a sentinel practitioners’ network composed of 23 general practitioners and 3 paediatricians representing respectively 3 % and 10 % of all physicians for each speciality. These physicians reported on a weekly basis the number of acute respiratory infection (ARI) and their total number of consultants. The number of ARI was extrapolated to the whole medical activity on the Island using the total number of consultants provided by social insurances’ data for the week considered. The number of consultants with A (H1N1) 2009 infection was obtained by applying the proportion of positivity for each week calculated on a random sample of patients with ARI tested by sentinel practitioners, to the total estimated number of consultants with ARI for the same week. At the end of the epidemic wave, a telephone survey has been conducted on a representative sample of the population to estimate the proportion of symptomatic non-consultants. 

The initial reproduction number (R_i_) was calculated following the intrinsic growth rate (r) method, assuming that the generation time follows a gamma distribution[2]. The sensitivity of R_i_ was tested to different estimates of r and to three recent estimates of the generation time: 1.9 days, σ = 0.893[2]; 2.7 days, σ = 1.1[3]; 2.8 days, σ = 1.319[4]. The herd immunity (HI) required to prevent a future epidemic on Réunion Island has been calculated assuming that the initial reproduction number is a good approximation of the basic reproduction number (Ri ≈ R0). In the absence of vaccination and assuming that immunity acquired previously was negligible, it can be calculated following the equation:

\begin{equation*}HI = 1 - \frac{1}{R_{i} }\end{equation*}

## Results

On Réunion Island, the pandemic wave was observed from week 30 and peaked on week 35 (Fig. 2).

**Figure:**
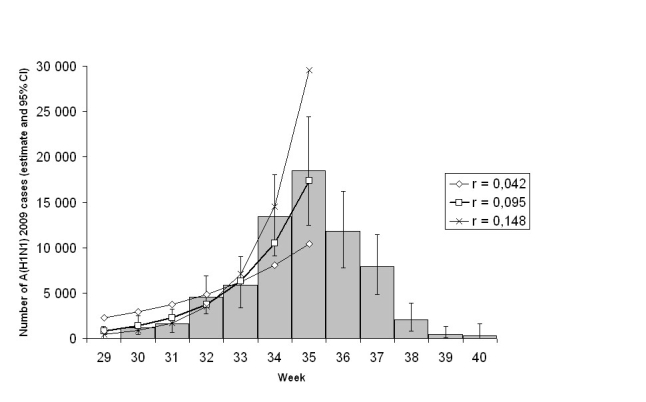


Figure 2: Weekly estimate of the number of influenza A pandemic H1N1 2009 incident cases from 13 July to 4 October 2009 and representation of the expected number of cases to the epidemic peak for three estimates of the intrinsic growth rate (r)

From week 30 to the end of the epidemic on week 38, the number of cases of A (H1N1) 2009 infections who have consulted a physician was estimated at 66,000 persons and the estimated cumulative attack rate for A(H1N1) 2009 infected consultants was 8.14 %. The rate of symptomatic non-consultants was 35.7 %.  Thus, the attack rate for A(H1N1) 2009 symptomatic infections was estimated to be 12.85 %. Figure 2 compares the calculated incidence for different values of r to the real epidemic growth. The result of sensitivity assessment gives a mean initial reproduction number of R_i_ = 1.26, ranging from 1.08 to 1.49 (Table 1).

Table 1: Sensitivity of the initial reproduction number to different estimates of intrinsic growth rate and generation time

**Table:** 

Generation time	Intrinsic growth rate			
	r = 0.042	r = 0.095	r = 0.148	Mean
1.9 days, σ = 0.893	R_i_ = 1.08	R_i_ = 1.19	R_i_ = 1.31	R_i_ = 1.19
2.7 days, σ = 1.1	R_i_ = 1.12	R_i_ = 1.29	R_i_ = 1.47	R_i_ = 1.29
2.8 days, σ = 1.319	R_i_ = 1.12	R_i_ = 1.31	R_i_ = 1.49	R_i_ = 1.31
Mean	R_i_ = 1.11	R_i_ = 1.26	R_i_ = 1.42	R_i_ = 1.26

The proportion of immune population (herd immunity) needed to stop the epidemic growth was estimated at 20 %, ranging from 7.4 % to 32.9 %.

## Discussion

These values for the reproduction number are among the lowest calculated so far[5]. They appear surprisingly low if one considers the alarming forecast used for pandemic preparedness. But this forecast was based on a retrospective assessment of the devastating health impact of the pandemic of 1918, which is far superior to that of this pandemic wave, at least regarding Réunion Island.

The level of herd immunity  needed to prevent another wave of infection with the same virus is slightly above the attack rate of 12.85 % calculated on Réunion Island. However, this attack rate underestimates probably the herd immunity acquired at the end of this pandemic wave because it does not take into account cases treated directly to hospital, nor the proportion of asymptomatic infections. Given the usual benignity of the disease observed on the Island, the proportion of asymptomatic infections could be around 30 %, as for seasonal influenza. Thus, although a new pandemic wave linked to the northern hemisphere epidemic during the austral summer cannot be totally excluded, it appears very unlikely, unless a significant viral mutation affects its antigenic characteristics.

The method used to estimate the reproduction number is very sensitive to the initial growth rate of the epidemic. To control the risk of error due to a possible underestimation of r, we tested the sensitivity of the estimate for different values of intrinsic growth rate. Furthermore, it should be noted that our estimate is very close to that obtained for the general population of Ontario with a completely different method based on a Monte-Carlo simulation[2]. 

This low value of the reproduction number may reflect a limited potential for transmission of the virus strain and / or some level of pre-existing herd immunity. It is also possible that the contact rate between persons was reduced during the epidemic wave, whether or not as a result of mitigation measures. 

Further studies are underway to assess the proportion of population that remains susceptible after this first wave in the event of a second wave.

## Funding information

Funded by the French Institute for Public Health Surveillance (Institut de veille sanitaire, InVS) as part of its surveillance mission ; InVS is funded by the French Ministry of Health 

## Competing interests

The authors have declared that no competing interests exist.
